# *Schistosoma japonicum* Infection Leads to the Reprogramming of Glucose and Lipid Metabolism in the Colon of Mice

**DOI:** 10.3389/fvets.2021.645807

**Published:** 2021-03-10

**Authors:** Xiaoying Yang, Weimin Ding, Xinyu Qian, Pengfei Jiang, Qingqing Chen, Xin Zhang, Yang Lu, Jiacheng Wu, Fenfen Sun, Zhihua Pan, Xiangyang Li, Wei Pan

**Affiliations:** ^1^Jiangsu Key Laboratory of Immunity and Metabolism, Department of Pathogen Biology and Immunology, Xuzhou Medical University, Xuzhou, China; ^2^National Experimental Teaching Demonstration Center of Basic Medicine, Xuzhou Medical University, Xuzhou, China; ^3^School of Life Sciences, Xuzhou Medical University, Xuzhou, China; ^4^The First Clinical Medical College, Xuzhou Medical University, Xuzhou, China; ^5^The Second Clinical Medical College, Xuzhou Medical University, Xuzhou, China

**Keywords:** *Schistosoma japonicum*, colon, soluble egg antigens, glucose and lipid metabolism, PTEN

## Abstract

The deposition of *Schistosoma japonicum* (*S. japonicum*) eggs commonly induces inflammation, fibrosis, hyperplasia, ulceration, and polyposis in the colon, which poses a serious threat to human health. However, the underlying mechanism is largely neglected. Recently, the disorder of glucose and lipid metabolism was reported to participate in the liver fibrosis induced by the parasite, which provides a novel clue for studying the underlying mechanism of the intestinal pathology of the disease. This study focused on the metabolic reprogramming profiles of glucose and lipid in the colon of mice infected by *S. japonicum*. We found that *S. japonicum* infection shortened the colonic length, impaired intestinal integrity, induced egg-granuloma formation, and increased colonic inflammation. The expression of key enzymes involved in the pathways regulating glucose and lipid metabolism was upregulated in the colon of infected mice. Conversely, phosphatase and tensin homolog deleted on chromosome ten (PTEN) and its downstream signaling targets were significantly inhibited after infection. In line with these results, *in vitro* stimulation with soluble egg antigens (SEA) downregulated the expression of PTEN in CT-26 cells and induced metabolic alterations similar to that observed under *in vivo* results. Moreover, PTEN over-expression prevented the reprogramming of glucose and lipid metabolism induced by SEA in CT-26 cells. Overall, the present study showed that *S. japonicum* infection induces the reprogramming of glucose and lipid metabolism in the colon of mice, and PTEN may play a vital role in mediating this metabolic reprogramming. These findings provide a novel insight into the pathogenicity of *S. japonicum* in hosts.

## Introduction

Schistosomiasis, a global neglected tropical disease (NTDS) caused by the organisms belonging to the genus *Schistosoma*, including *S. japonicum, S. mansoni*, and *S. haematobium*, is considered the second-most socially and economically devastating parasitic disease (1). As the most problematic of human helminthiases, schistosomiasis threatens more than 800 million people and affects more than 250 million people worldwide (2).

Migrating parasite eggs are regarded as the major pathogenic factor of schistosomiasis, contributing to the formation of tissue lesions, particularly in the intestinal tract (3). A large number of schistosome eggs trapped in the intestinal wall lead to the formation of egg granulomas and result in symptoms such as diarrhea, inflammation, fibrosis, hyperplasia, and polyposis (4). The immune mechanism of intestinal lesions in schistosomiasis has been studied previously (5). Polarized T-helper 2 (Th2)-mediated immune responses are responsible for the development of chronic intestinal pathologies induced by schistosome eggs and soluble egg antigens (SEA) (6). Many cell types, such as basophils, B cells, regulatory T cells (Treg), and dendritic cells (DCs), have been implicated in the induction or maintenance of Th2 responses against eggs and SEA (7–9). Furthermore, some independent studies in humans and rodents have demonstrated that fecal microbiome differences linked to host immune-regulation or inflammation are associated with parasitic infection (5, 10, 11).

Recently, many studies have focused on the reverse effect of schistosome infection on metabolism-related diseases and found that chronic *S. japonicum* infection or treatment with SEA could regulate host metabolism via Th2 responses (12, 13). As an important organ for digestion, absorption, and metabolism of dietary nutrients, the glucose and lipid metabolism in the intestinal tract is important for host health. However, the potential effect of schistosome infection on intestinal glucose and lipid metabolism remains unclear.

Phosphatase and tensin homolog deleted on chromosome ten (PTEN) is an important member of the protein tyrosine phosphatase (PTP) gene family (14). PTEN is a tumor suppressor gene and regulates cell metabolism (15). For instance, PTEN overexpression could inhibit glycolysis in tumor cells (16). A previous study showed that hepatic PTEN-knockout increased the triglyceride (TG) content, reduced apolipoprotein B (ApoB) protein mass, and promoted fatty liver development (17). We previously reported that the decreased PTEN expression is associated with the metabolic reprogramming events in hepatic fibrosis induced by *S. japonicum* (18). However, whether PTEN and its downstream metabolic pathway participate in the formation of intestinal lesions induced by *S. japonicum* remains elusive.

In the present study, a murine model of *S. japonicum* cercaria infection and an *in vitro* model of SEA treated CT-26 cells were used to explore the possible role of schistosome infection in intestinal glucose and lipid metabolism. We found that *S. japonicum* infection induced the reprogramming of glucose and lipid metabolism in the intestine of mice, which accompanied with the inactivation of PTEN pathway. Consistent with this, SEA treatment could mimic similar metabolic alterations in CT-26 cells that were inhibited by PTEN overexpression. These findings might provide a novel insight on the formation of intestinal lesions and developing a metabolism-targeted strategy for the treatment of schistosomiasis.

## Materials and Methods

### Parasite Preparation and Animal Studies

The cercariae of *S. japonicum* (Chinese strain) were obtained from freshwater snails (*Oncomelania hupensis*) that were provided by the National Institute of Parasitic Diseases, Chinese Center for Disease Control and Prevention (Shanghai, China). Female BALB/c mice were obtained from Shanghai Laboratory Animal Center (SLAC, Shanghai, China) and housed and maintained with a 12 h light/dark photoperiod and *ad libitum* access to water and food. Then, mice were randomly divided into 2 groups: control group and model group. In the model group, mice were percutaneously infected with 20 cercariae of *S. japonicum*. At 9 weeks post-infection, mice were euthanized by cervical dislocation. The intestinal tissues were collected and stored at−80°C for further analyses.

### Preparation of SEA

Eggs were obtained from the livers of mice infected by *S. japonicum* for 9 weeks, and SEA were prepared as previously described (19). Briefly, the livers of infected mice were collected and cut into a number of pieces. Subsequently, the pieces were homogenized in LPS-free PBS on ice, filtered, washed, and centrifuged at 12,000 rpm for 15 min. Purified eggs were suspended in pre-cooled PBS containing 1 mM PMSF (Roche Diagnostics) and 2 μg/mL Leupeptin (Sigma) and homogenized on ice using a homogenizer (VirTis Co). The suspension was frozen/thawed several times and centrifuged at 12,000 rpm for 30 min at 4°C. A 0.22 μm filter was used to pass the supernatant and obtain SEA. The protein concentration of SEA was determined using the BCA Protein Assay Kit (Beyotime Biotech, Beijing, China).

### Histopathological Analysis

For histopathological analysis, intestinal tissues were fixed in 4% paraformaldehyde, embedded in paraffin, and divided into sections of 5 μm thickness. Then, the sections were stained with hematoxylin and eosin (H&E) according to the standard procedure. All sections were imaged under a microscope (OLYMPUS IX51), and digital photographs were captured for further analysis.

### Cell Lines and *in vitro* Treatment

Mouse colon cancer cell line CT-26 was purchased from Shanghai Cell Bank, Chinese Academy of Sciences and maintained in our lab. Cells (1 × 10^6^/mL) were seeded in 6-well plates in DMEM (glutamine, high glucose) supplemented with penicillin (100 units/mL), streptomycin (100 μg/mL) and 10% heat-inactivated FBS at 37°C with 5% CO _2_ in a humidified atmosphere. For treatment with SEA, cells were incubated with SEA (10 μg/mL) or PBS for 24 h. For co-treatment experiments, 24 h after transfection with PTEN plasmid, cells were incubated with SEA (10 μg/mL) or PBS for another 24 h. Cells were then harvested for further analysis.

### Cell Transfection

To overexpress PTEN, the CT-26 cells were transfected with Flag-PTEN using Lipofectamine™ 2000 (Invitrogen) according to the manufacturer's instructions. The Flag-tagged full-length mouse PTEN gene complementary DNA (cDNA) in pECMV-Myc-Flag vector (Shanghai, China) was used for transfection with a plasmid encoding an Ampicillin gene. Cells transfected with only the pECMV-Myc-Flag plasmid were used as control. After 24 h of culture, the CT-26 cells were respectively transfected with 2 μg *of plasmids* for 24 h.

### Western Blot Analysis

Western blot assays were performed as described previously (20). Total proteins were extracted from the pulverized intestine or cultured CT-26 cells in cell lysis buffer, and the concentration was determined using a BCA protein concentration assay kit (Beyotime Biotech, Beijing, China). A total of 40–80 μg of protein was resolved using 10 % SDS-PAGE gel, and separated proteins were electrotransferred to polyvinylidene difluoride membranes using a Bio-Rad electrophoresis system (Hercules, CA, USA). Western blot assays were performed using indicated primary antibodies specific for Occludin-2 (#48120, Cell Signaling Technology, USA, 1: 1000 dilution), ZO-1 (#8193, Cell Signaling Technology, USA, 1: 1000 dilution), the p85α subunit of Phosphatidylinositol 3-kinase (PI3K-p85α) (ab191606, abcam, UK, 1: 1000 dilution), p-AKT(ser473) (ab81283, abcam, UK, 1: 1000 dilution), p-AKT(ser129) (ab133458, abcam, UK, 1: 1000-1:10000 dilution), Total AKT(ab179463, abcam, UK, 1: 10000 dilution), β-actin (ab119716, abcam, UK, 1: 5000/1: 2000 dilution), PKM2 (#4053, Cell Signaling Technology, USA, 1: 1000 dilution), PGC-1α (ab54481, abcam, UK, 1: 1000 dilution), PPARα (WL00978, wanleibio, China, 1: 500-1: 1000 dilution), CPT-1a (#12252, Cell Signaling Technology, USA, 1: 1000 dilution) and SREBP (WL02093, wanleibio, China, 1: 1000-1: 1500 dilution). The immune reaction was performed using Clarity™ ECL western blot substrate (Bio-Rad, USA) and visualized using ChemiDoc Touch imaging system (Bio-Rad, USA).

### Quantitative Real-Time PCR (qPCR)

Total RNA was extracted with TRIzol (Termo Fisher Scientifc, USA) from the pulverized colon or cultured CT-26 cells. Then 1 μg RNA for each sample was reverse-transcripted to cDNA using a high-capacity cDNA reverse transcription kit (Takara, Japan). qPCR was performed using the LightCycler® 480II detection system (Roche Applied Science, Penzberg, Germany). All primers are listed in Table 1. All experiments were performed in triplicate, and the relative expression of related genes was indicated by comparative cycling threshold (Ct) value normalized against an endogenous reference (β-actin) using the 2^−ΔΔ*Ct*^ method.

**Table 1:** The real-time RT-PCR primers used in the study.

**Primer names**	**Sequences (5′ to 3′)**
MCAD	Forward:5′-TAACATACTCGTCACCCTTC-3′
	Reverse:5′-ATGCCTGTGATTCTTGCT-3′
CYP4A10	Forward:5′-GCAAACCATACCCAATCC-3′
	Reverse:5′-TCCCAAGTGCCTTTCCTA-3′
L-FABP	Forward:5′-TTGACGACTGCCTTGACT-3′
	Reverse:5′-GCCAGGAGAACTTTGAGC-3′
ACC1	Forward:5′-TGCTGGATTATCTTGGCTTCA-3′
	Reverse:5′-CCCGTGGGAGTAGTTGCTGTA-3′
FAS	Forward:5′-TCGGAGACAATTCACCAAACC-3′
	Reverse:5′-AGCCATCCCACAGGAGAAACC-3′
SCD1	Forward:5′-CTTCCTCCTGAATACATCCCTCC-3′
	Reverse:5′-CTCCATCCCATCTAGCACAACCT-3′
PPARα	Forward:5′-CTGTCGGGATGTCACACAATGC-3′
	Reverse:5′-TCTTTCAGGTCGTGTTCACAGGTAA-3′
G6pc	Forward:5′-TGGACGGAAGCAATTTTTCA-3′
	Reverse:5′-GTCTCACAGGTGACAGGGAAC-3′
CPT-1a	Forward:5′-TATGGTCAAGGTCTTCTCGGGTCG-3′
	Reverse:5′-AGTGCTGTCATGCGTTGGAAGTCTC-3′
GLUT2	Forward:5′-TCAGAAGACAAGATCACCGGA-3′
	Reverse:5′-GCTGGTGTGACTGTAAGTGGG-3′
GLUT4	Forward:5′-GATTCTGCTGCCCTTCTGTC-3′
	Reverse:5′-ATTGGACGCTCTCTCTCCAA-3′
HIF-1α	Forward:5′-GTCGGACAGCCTCACCAAACAG-3′
	Reverse:5′-TAGGTAGTGAGCCACCAGTGTCC-3′
CS	Forward:5′-CGAATTTGAAAGATGTACTGAGC-3′
	Reverse:5′-CTTAGGCAGCATTTTCTGGC-3′
PFK	Forward:5′-GCCACTAAGATGGGTGCTAAGG-3′
	Reverse:5′-CGTACTTGGCTAGGATTTTGAGG-3′
PK	Forward:5′-CAGCCATGGCTGACACCTTC-3′
	Reverse:5′-GGATCAGATGCAAAGCTTTCTG-3′
IDH3G	Forward:5′-GAGTGGTGACCCGGCAC-3′
	Reverse:5′-TCCATCACCCAGTTTCATGATG-3′
IL-1β	Forward:5′-TCCAGGGACAGGATATGGAG-3′
	Reverse:5′-TCTTTCAACACGCAGGACAG-3′
IL-6	Forward:5′-CCACGGCCTTCCCTAC-3′
	Reverse:5′-AAGTGCATCATCGTTGT-3′
IL-10	Forward:5′-GCTCCAGAGCTGCGGACT-3′
	Reverse:5′-TGTTGTCCAGCTGGTCCTTT-3′
TNF-α	Forward:5′-CATCTTCTCAAAATTCGAGTGACAA-3′
	Reverse:5′-TGGGAGTAGACAAGGTACAACCC-3′
TGF-β	Forward:5′-CTGGATACCAACTACTGCTTCAG-3′
	Reverse:5′-TTGGTTGTAGAGGGCAAGGACCT-3′
MCP1	Forward:5′-AGAGAGCCAGACGGAGGAAG-3′
	Reverse:5′-GTCACACTGGTCACTCCTAC-3′
PTEN	Forward:5′-AATTCCCAGTCAGAGGCGCTATGT-3′
	Reverse:5′-GATTGCAAGTTCCGCCACTGAACA-3′
Pck1	Forward:5′-AGCCTCGACAGCCTGCCCCAGG-3′
	Reverse:5′-CCAGTTGTTGACCAAAGGCTTTT-3′

### Statistical Analysis

Data were analyzed using GraphPad Prism software 5.0 and are presented as mean ± SEM. Statistical significance was determined using the unpaired 2-tailed Student's *t*-test for a single variable and one-way analysis of variance (ANOVA) followed by the *post hoc* Tukey test for multiple comparisons. Values with *p* < 0.05 were considered statistically significant.

## Results

### Comparison of the Colonic Pathology in *S. japonicum*-Infected Mice vs. Uninfected Control Mice

**Figure 1:**
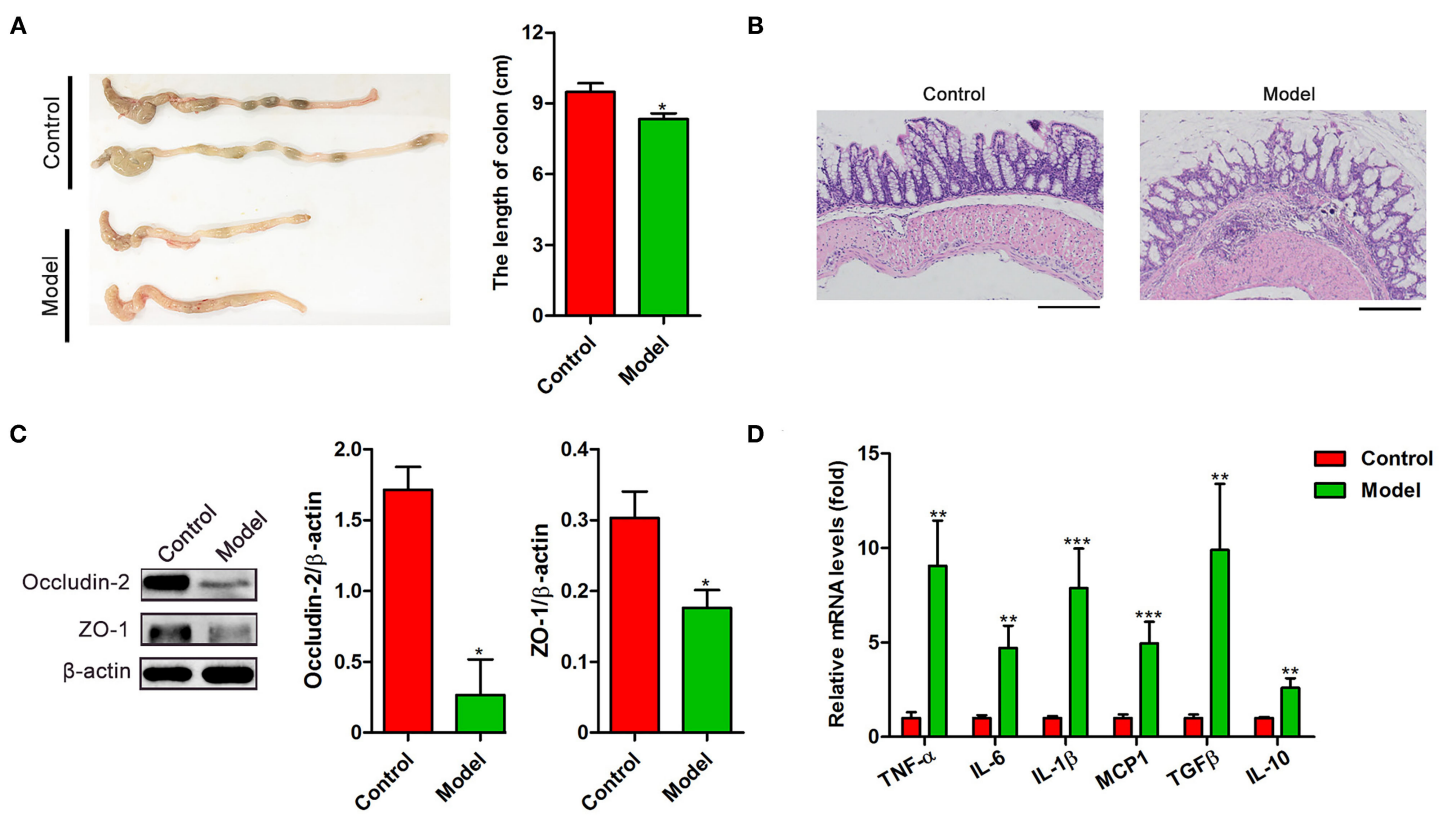
The intestinal pathological analysis and the cytokines expression of BALB/c mice infected with *S. japonicum*. Intestinal tissues were obtained from control mice or mice infected with *S. japonicum* for 9 weeks. **(A)** Representative photographs of colons (Left) and the length comparison of colon (right, *n* = 15). **(B)** Representative images of H&E stained colon. **(C)** The protein expression levels of occludin-2 and ZO-1(*n* = 4). Data are expressed as mean ± standard error of means. Similar results were observed in three independent experiments. **(D)** The expression of genes related inflammatory (TNF-α, IL-6, IL-1β, and MCP1) and anti-inflammatory cytokines (TGF-β and IL-10) (*n* = 4). Values are mean ± standard error of means. (Student's *t*-test: *p < 0.05, **p < 0.01, ***p < 0.001).

To investigate the intestinal pathological changes after *S. japonicum* infection, 15 BALB/c mice (Model) were infected with 20 *S. japonicum* cercariae, while 15 uninfected gender and age-matched mice were used as controls. The colonic lesions were evaluated after 9 weeks post-infection. As shown in Figure 1A, compared to the control mice, the length of the colon in the infected mice was significantly shortened (*p* < 0.05). H&E staining showed that atypical changes such as irregular colonic glands, decreased goblet cells in the mucosa, and the presence of egg granulomas in the submucosa of infected mice indicated significant damage of the intestinal function (Figure 1B). In line with these changes, the colonic expression of occludin-2 and Zonula occludens 1 (ZO-1), the tight junction proteins crucial for the maintenance of intestinal barrier integrity, was decreased in infected mice (both *p* < 0.05, Figure 1C). Furthermore, we observed that *S. japonicum* infection increased the mRNA levels of proinflammatory cytokines TNF-α, IL-6, IL-1β, and Monocyte chemotactic protein-1 (MCP-1) along with anti-inflammatory cytokines Transforming growth factor-β (TGF-β) and IL-10 (both *p* < 0.01, Figure 1D) in the colon. These results suggest that *S. japonicum* infection could cause colonic injury in mice.

### Expression Profile of Colonic Genes Regulating Glucose and Lipid Metabolism During *S. japonicum* Infection

**Figure 2:**
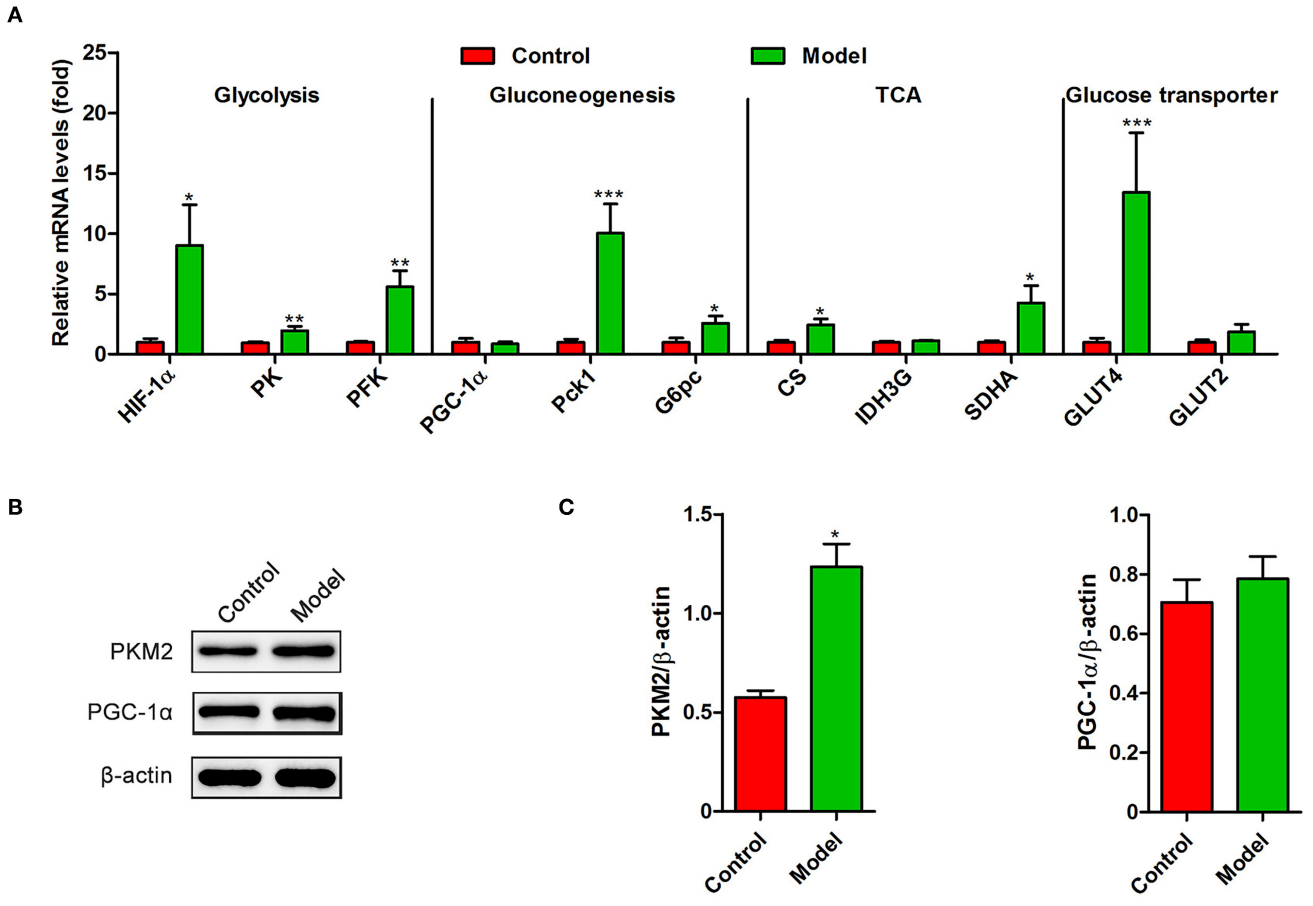
The expression profiling of glucose metabolism-related genes in the colon of *S. japonicum*-infected mice. Intestinal tissues were obtained from control mice or mice infected with *S. japonicum* for 9 weeks. **(A)** The mRNA levels of glycolysis-related genes (HIF-1α, PK and PFKFB3), gluconeogenesis-related genes (PGC-1α, Phosphoenolpyruvate carboxykinase 1 and G6pc), TCA-related genes (CS, SDHA, and IDH3G) and Glucose transporter-related genes (GLUT4 and GLUT2). **(B,C)** The protein expression level of PKM2 and PGC-1α. Data are expressed as mean ± standard error of means (*n* = 4). Similar results were observed in three independent experiments. (Student's *t*-test: *p < 0.05, **p < 0.01, ***p < 0.001).

**Figure 3:**
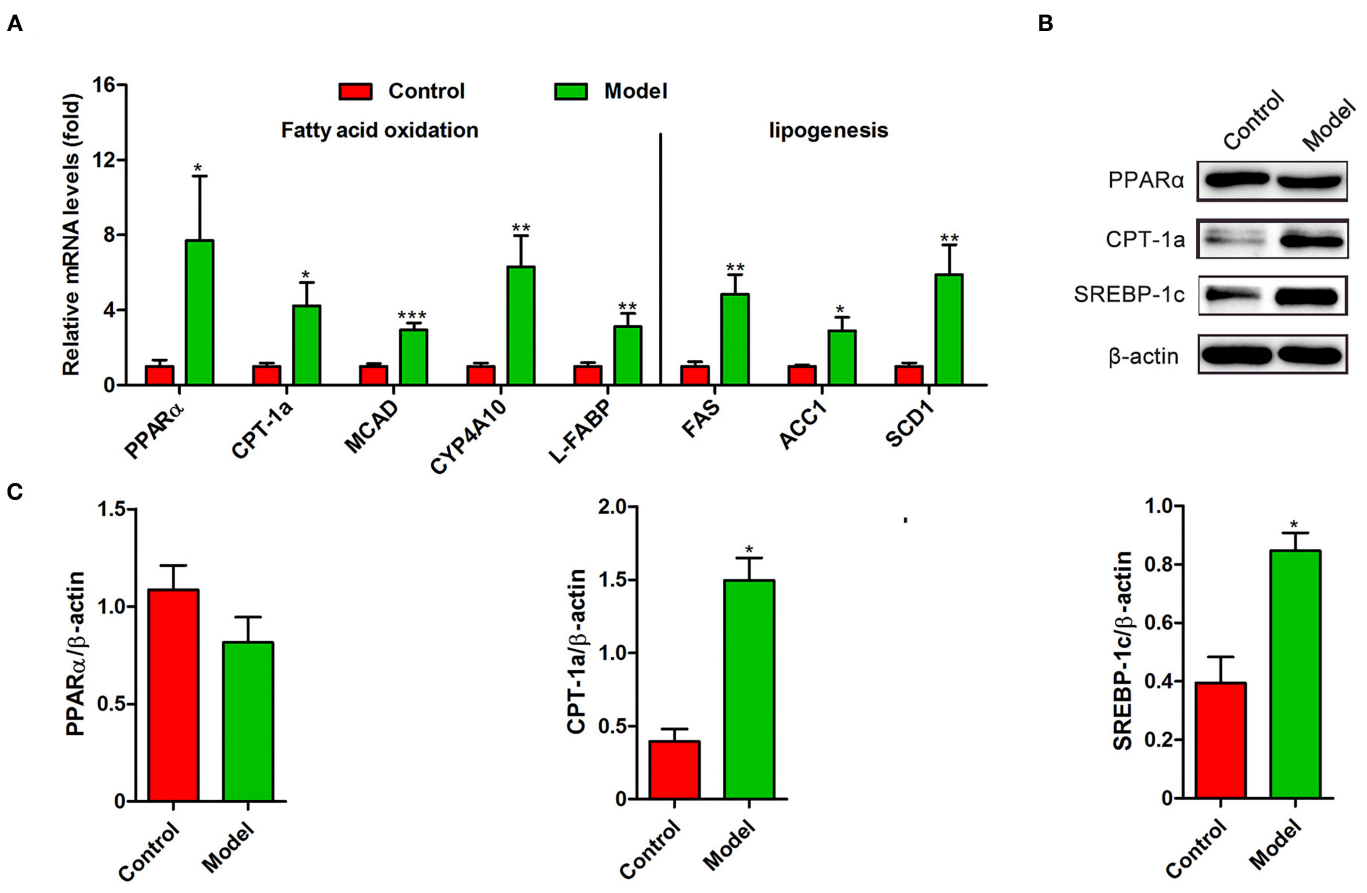
The expression profiling of lipid metabolism-related genes in the colon of *S. japonicum*-infected mice. **(A)** The mRNA levels of genes related to fatty acid (FA) oxidation (PPARα, CTP-1a, MCAD, CYP4A10 and L-FABP) and FA synthesis (FAS, ACC1 and SCD1) (*n* = 4). **(B,C)** The protein expression levels of PPARα, CTP-1a and SREBP-1c (*n* = 4). Data are expressed as mean ± standard error of means. Similar results were observed in three independent experiments. (Student's *t*-test: *p < 0.05, **p < 0.01, ***p < 0.001).

Recently, several studies have demonstrated that *S. japonicum* infection could regulate hepatic glycolipid metabolism and inverse metabolism-related diseases (12, 20). Here, we profiled the changes in the expression of colonic genes involved in glucose and lipid metabolism caused by *S. japonicum* infection. As shown in Figure 2A, compared with the control mice, *S. japonicum* infected mice showed a higher expression of glycolytic pathway related proteins (Hypoxia-inducible factor 1α, HIF-1α; Pyruvate kinase isozyme type M2, PKM2), gluconeogenesis genes (Phosphoenolpyruvate carboxykinase 1, Pck1, and Glucose-6 phosphatase, G6pc), tricarboxylic acid cycle (TCA) enzymes (Citrate synthase, CS; Succinate dehydrogenase complex flavoprotein subunit A, SDHA), and glucose transporter 4 (GLUT4) (All *p* < 0.05). Other genes associated with these signaling pathways, such as Peroxisome proliferator-activated receptor-γ coactivator-1α (PGC-1α), Isocitrate Dehydrogenase 3 (IDH3G), and Glucose transporter 2 (GLUT2), did not demonstrate any remarkable changes. Consistent with these results, the protein expression of PKM2 was significantly upregulated in infected mice, and the PGC-1α protein expression was similar between control and *S. japonicum* infected mice (Figures 2B,C). Besides, *S. japonicum* infection increased the mRNA expression of fatty acid oxidation genes (Peroxisome proliferator-activated receptor alpha, PPARα; Carnitine palmitoyl transferase 1a, CTP-1a; Medium-chain acyl-CoA Dehydrogenase, MCAD; Cytochrome P450 proteins 4A10, CYP4A10 and Liver-fatty acid binding protein, L-FABP) and lipogenesis-related genes (Fatty acid synthase, FAS; Acetyl coenzyme A carboxylase 1, ACC1 and Stearoyl-CoA desaturase 1, SCD1) in the colon (All *p* < 0.05, Figure 3A). In line with this, the colonic protein expressions of CTP-1a and Sterol regulatory element-binding protein-1c (SREBP-1c) were significantly upregulated after *S. japonicum* infection (Figures 3B,C). However, *S. japonicum* infection did not affect PPARα colonic protein expression (Figures 3B,C). Together, these results indicate that *S. japonicum* infection promotes the expression of genes involved in *glucose and lipid* metabolism in the colon.

### Inhibition of PTEN/PI3K/AKT Pathway in the Intestine Mediated by *S. japonicum*

**Figure 4:**
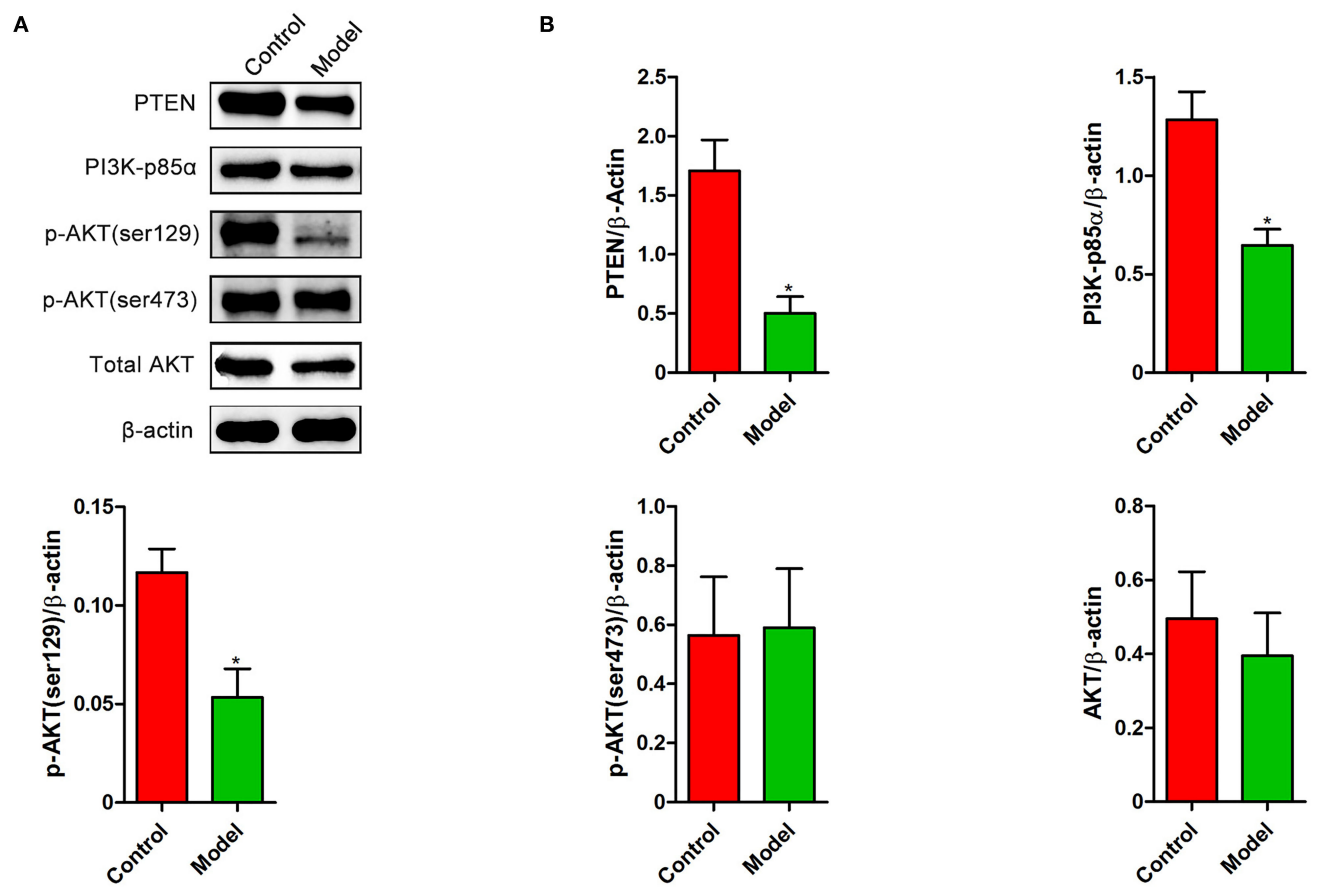
The expression changes of PTEN/PI3K/AKT pathway in the colon of *S. japonicum*-infected mice. Intestinal tissues were obtained from control mice or mice infected with *S. japonicum* for 9 weeks. **(A,B)** The protein expression levels of PTEN, PI3K (p85α subunits), p-AKT (ser129), p-AKT (ser473), and total AKT (*n* = 4). Data are expressed as mean ± standard error of means. Similar results were observed in three independent experiments. (Student's *t*-test: *p < 0.05).

We next investigated whether PTEN participates in the formation of intestinal lesion induced by *S. japonicum*. A decrease in the levels of PTEN was observed in the colon after *S. japonicum* infection (Figure 4). The expression of PI3K (p85α subunit) and the phosphorylated (Ser129) form of AKT (Figures 4A,B), which are important downstream signaling targets of PTEN, were significantly downregulated. However, no remarkable differences were observed in the phosphorylated (Ser473) form of AKT and total AKT protein levels in the colon between control mice and *S. japonicum* infected mice. Overall, these results indicate that the colonic PTEN/PI3K/AKT pathway was inhibited by *S. japonicum* infection.

### The effect of SEA and PTEN Overexpression on Glucose and Lipid Metabolism *in vitro*

**Figure 5:**
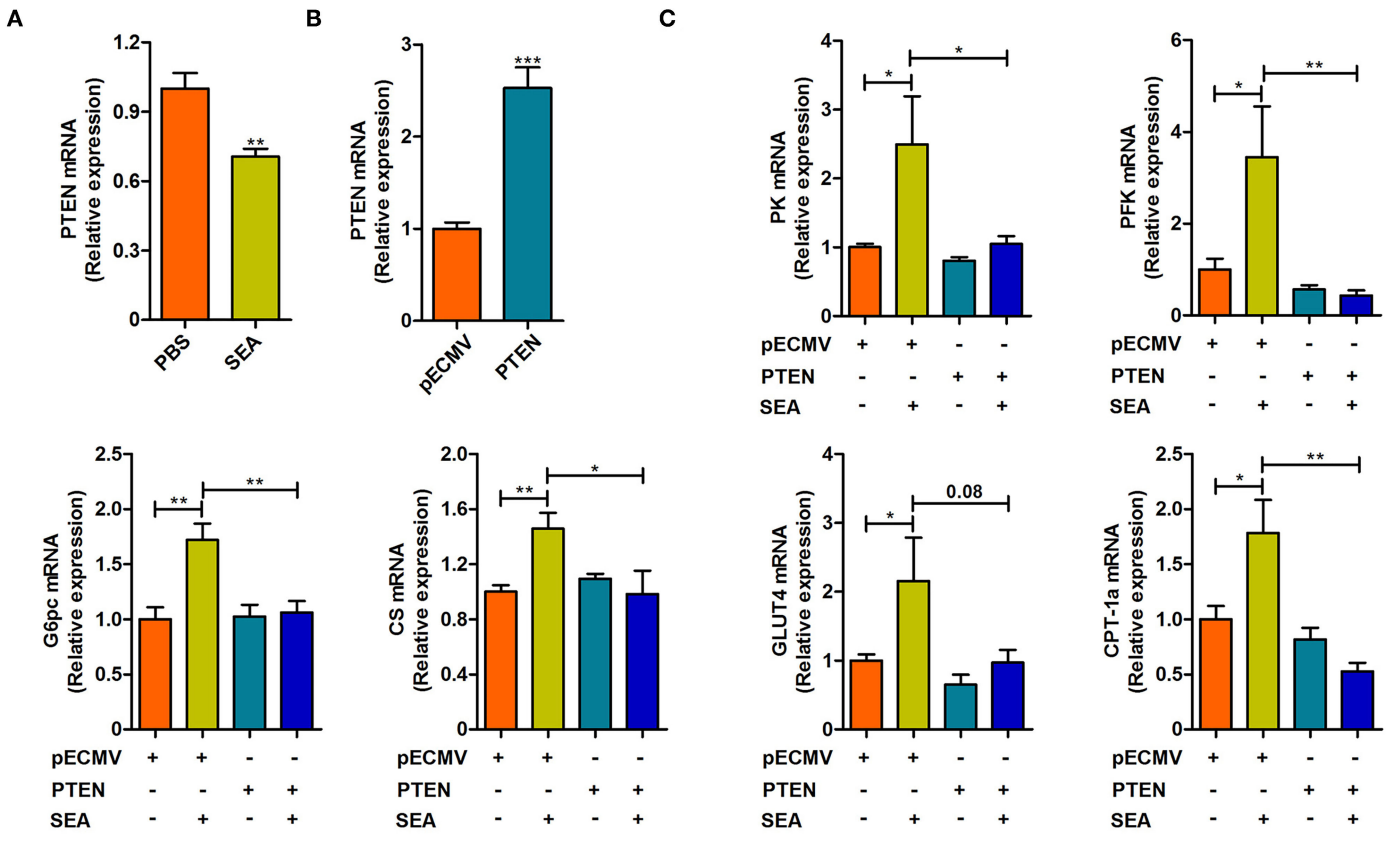
The effect of SEA treatment and PTEN overexpression on the reprogramming of glucose and lipid metabolism in CT-26 cells. **(A)** The mRNA level of PTEN in CT-26 cells stimulated with SEA (10 μg/ml) for 24 h prior to harvest. **(B)** The mRNA level of PTEN in CT-26 cells transfected with PTEN plasmid for 24 h prior to harvest. **(C)** the mRNA levels of metabolic genes in CT-26 cells transfected with indicated plasmids for 24 h, followed by treatment with SEA (10 μg/ml) for 24 h prior to harvest. Data are mean ± standard error of means. (one-way ANOVA: *p < 0.05, **p < 0.01, ***p < 0.001).

SEA secreted by the eggs are the primary pathogenic components of *S. japonicum* infection (20). We next stimulated CT-26 cells with SEA *in vitro* to detect the mechanisms underlying the aforementioned phenotypes. As shown in Figure 5, the SEA stimulation was associated with a decrease in the expression of PTEN (*p* < 0.05, Figures 5A,B), in accordance with the results observed under *in vivo* conditions. Meanwhile, the expression of PK, PFK, G6pc, CS, GLUT4, and Carnitine palmitoyl transferase 1a (CPT-1a) was significantly upregulated after exposure to SEA compared to that observed in the control group (all *p* < 0.05), which was consistent with our results observed under *in vivo* conditions. Importantly, overexpression of PTEN prevented SEA-mediated increase in the mRNA levels of aforementioned glucose and lipid metabolic genes, except GLUT4 (all *p* < 0.05, Figure 5C). Collectively, these results indicate that SEA may participate in the regulation of intestinal glucose and lipid metabolism after *S. japonicum* infection, and these effects can be attributed to the inhibition of PTEN.

## Discussion

The present study demonstrated that *S. japonicum* infection could impair colonic integrity, increase colonic inflammation, and induce the reprogramming of glucose and lipid metabolism in the colon of mice. The mechanism of metabolic reprogramming was demonstrated in CT-26 cells with SEA stimulation, which was mediated by PTEN inhibition. To our knowledge, this is the first study to investigate metabolic reprogramming in the intestine of mice infected with *S. japonicum*, which may provide a new perspective for understanding the formation of intestinal lesions induced by *S. japonicum* infection.

Schistosomiasis is a widely distributed chronic disease that threatens human health and social and economic development. The formation of intestinal lesions caused by the migration of parasite eggs is one of the main pathological characteristics of this disease (4). In the present study, we observed that the length was shorter and was accompanied by colonic epithelial edema. In response to these changes, we observed intestinal granulomas, impaired intestinal integrity, and increased production of pro-inflammatory cytokines in infected mice. A previous study showed that schistosomiasis could stimulate type 2 immune response (8, 21). Our results showed that the expression of anti-inflammatory cytokines (TGF-β and IL-10) in infected mice was higher than that in control mice, in agreement with previous reports. All of these results indicated that *S. japonicum* infection damaged the intestinal structure.

As an emerging field, immunometabolism investigates the interface between the metabolism and immune response and explores how metabolic pathways, especially glucose and lipid metabolism, govern the phenotype of the immune response. So far, many metabolic processes, as such glycolysis, the Krebs cycle, and fatty acid metabolism have been found to play a vital role in immune response (22). Activating the glycolytic pathway could shift immune cells (macrophages, Treg cells, B cells, and DC cells) from an anti-inflammatory status to a pro-inflammatory state. A shift in cell metabolism leading to fatty acid oxidation and oxidative phosphorylation demonstrates an opposite effect (23–25). The previously mentioned immune cells are involved in the induction of immune responses against schistosomiasis (7–9, 20). In accordance with the upregulation of intestinal pro-inflammatory and anti-inflammatory cytokines, the glycolysis, TCA, and fatty acid oxidation pathways were significantly activated in infected mice.

Recently, the reverse relationship between schistosome infection and metabolic syndrome has received attention (12, 20). An epidemiological study revealed that schistosome infection is negatively correlated with metabolic diseases in humans (26). Meanwhile, animal studies confirmed that chronic *S. japonicum* infection could improve hepatic insulin sensitivity and glucose metabolism in mice (13). The levels of lipoproteins were markedly suppressed in the serum of infected mice (27). Acute *S. mansoni* infection impaired lipid absorption in the gut of mice, and chronic *S. mansoni* infection reduced fat mass in obese mice (28). Our previous study showed that *S. japonicum* infection decreased the levels of serum glucose and triacylglycerol in mice and regulated hepatic glucose and lipid metabolism (18). The intestine is an important organ for digestion, absorption, and metabolism of dietary nutrients, whose metabolism is important for host health (19). Besides the glycolysis, TCA, and fatty acid oxidation pathways, we also detected alterations in gluconeogenesis, glucose transport, and fatty acid synthesis in the intestine. All of these pathways involved in glucose and lipid metabolism were enhanced based on the expression of rate-limiting enzymes in the infected group. These changes may be attributed to the compensatory effect of liver and intestine damage. Liver fibrosis induced by S. *japonicum* infection impaired its metabolic function (29). To meet the energy needs of the host, the intestinal function was enhanced to compensate for the damage of the liver. The enhanced metabolism may boost the intestinal immunity.

SEA released by *S. japonicum* eggs can recruit inflammatory and immune cells to the lesion region, causing egg granuloma formation and worsening the pathology of schistosomiasis (30). SEA treatment can improve insulin sensitivity and reduce fat mass in obese mice model developed by the administration of a high-fat diet, similar to the effects of chronic *S. japonicum* infection (31). *In vitro* stimulation by SEA caused the reprogramming of glycolipid metabolism in macrophages, which could significantly reduce the expression of Acc mRNA in hepatocytes (20). Our previous study showed that SEA stimulation directly upregulated the expression of GLUT4, MCAD, ACC1, FAS, CYP4A10, and CS in hepatocytes. Therefore, in some respects, SEA may be a crucial contributor to the reprogramming of glucose and lipid metabolism in lesions induced by S. *japonicum*. In line with this hypothesis, we observed that SEA treatment directly increased the mRNA expression of genes associated with glucose and lipid metabolism in CT-26 cells.

PTEN is an anti-oncogene that has a close relationship with liver fibrosis (32) and plays an essential role in immunity (33). According to our previous study, the downregulation of PTEN was linked with the changes of glucose and lipid metabolism in *S. japonicum*-induced liver fibrosis (18), and treatment with polyene phosphatidylcholine (PTEN agonist) showed the reversal of the alterations in glycolysis and fatty acid oxidation pathways induced by SEA. Therefore, PTEN may play an essential role in the reprogramming of metabolism in *S. japonicum*-induced tissue lesions. In the present study, we observed that the PTEN/PI3K/AKT pathway was inhibited in the infected group, and SEA stimulation decreased the mRNA levels of PTEN in CT-26 cells. Moreover, overexpression of PTEN reversed the changes in genes involved in the glucose and lipid metabolism in CT26 cells. These results suggested that the PTEN/PI3K/AKT pathway may play a role in the intestinal pathological metabolic changes in schistosomiasis.

## Conclusions

In summary, the present study identified the expression of genes involved in the intestinal glucose and lipid metabolism after *S. japonicum* infection and verified that SEA initiated a cellular metabolic response via the inhibition of PTEN in enterocytes. Our findings provide a novel insight into the pathogenesis of *S. japonicum* with respect to the formation of intestine lesions, and investigation of PTEN and its role in schistosomiasis may be a new direction for understanding metabolic reprogramming in schistosomiasis.

## Data Availability Statement

The original contributions generated for the study are included in the article, further inquiries can be directed to the corresponding author/s.

## Ethics Statement

The animal study was reviewed and approved by The Ethics Committee of Xuzhou Medical University (Xuzhou, China, SCXK (FS) 2015-0009).

## Author Contributions

XY, WP, and FS: conceived and designed the experiments. WD, XQ, XZ, QC, YL, and JW: performed the experiments. XY, WD, and XQ: analyzed the data. XY, WP, FS, XL, and ZP: contributed reagents, materials, and analysis tools. WD, XQ, PJ, and XY: wrote the manuscript. All authors have read and approved the manuscript.

## Conflict of Interest

The authors declare that the research was conducted in the absence of any commercial or financial relationships that could be construed as a potential conflict of interest.
